# Radiomic white matter parameters of functional integrity of the corticospinal tract in high-grade glioma

**DOI:** 10.1038/s41598-024-63813-2

**Published:** 2024-06-05

**Authors:** Tim Wende, Erdem Güresir, Johannes Wach, Martin Vychopen, Anastasia Hoffmann, Gordian Prasse, Florian Wilhelmy, Johannes Kasper

**Affiliations:** 1 Department of Neurosurgery, University Hospital Leipzig, Liebigstr. 20, 04103 Leipzig, Germany; 2 Institute of Neuroradiology, University Hospital Leipzig, Leipzig, Germany

**Keywords:** CNS cancer, Prognostic markers, White matter disease, Brain, Magnetic resonance imaging, Brain imaging

## Abstract

Tractography has become a widely available tool for the planning of neurosurgical operations as well as for neuroscientific research. The absence of patient interaction makes it easily applicable. However, it leaves uncertainty about the functional relevance of the identified bundles. We retrospectively analyzed the correlation of white matter markers with their clinical function in 24 right-handed patients who underwent first surgery for high-grade glioma. Morphological affection of the corticospinal tract (CST) and grade of paresis were assessed before surgery. Tractography was performed manually with MRTrix3 and automatically with TractSeg. Median and mean fractional anisotropy (FA) from manual tractography showed a significant correlation with CST affection (p = 0.008) and paresis (p = 0.015, p = 0.026). CST affection correlated further most with energy, and surface-volume ratio (p = 0.014) from radiomic analysis. Paresis correlated most with maximum 2D column diameter (p = 0.005), minor axis length (p = 0.006), and kurtosis (p = 0.008) from radiomic analysis. Streamline count yielded no significant correlations. In conclusion, mean or median FA can be used for the assessment of CST integrity in high-grade glioma. Also, several radiomic parameters are suited to describe tract integrity and may be used to quantitatively analyze white matter in the future.

## Introduction

White matter tracts in the brain have always been of interest to neuroscientists, who studied both their anatomy and their function^1,2^. Due to their organization in networks, connectomic analyses have been carried out to further understand brain function and plasticity^3^. While these approaches carry great potential to unravel new insights, it is of utmost importance to choose physical quantities that actually describe the connections that are made by axons in white matter tracts between different brain regions. However, little data exists to address this question, especially in the presence of brain tumors^4,5^.

Information in the brain travels along axons on an electrical basis and is transferred in synapses, which are mostly chemical^6^. When a larger number of axons travels together, they form a white matter tract, which gains its volume due to the surrounding myelin sheaths. These sheaths electrically isolate the axons and form a diffusion barrier that causes diffusion tensors to point in the tract direction when measured with diffusion tensor imaging (DTI) sequences in magnetic resonance imaging (MRI). Diffusion tensors can be used to create tractograms by using highly sophisticated algorithms that are able to consider crossing fibers as well as the presence of tumors, while still relying on expert interaction in the workflow^7^. Further, diffusion markers like fractional anisotropy (FA) can be calculated per voxel. FA is a measure of the directedness of diffusion and can take on values between 0 and 1. As of now, it is the most robust marker of white matter integrity^8–14^.

Tractograms usually consist of streamlines, describing the probability that a white matter tract is present along their coordinates^12^. This spatial information can then be used to define volumes of the depicted tracts^15^. While a great number of connectomic studies use streamline count or its derivates to measure structural connectivity, its biological relevance has not yet been proven^16^. On the other hand, diffusion measures like the mean FA of voxels in a tract volume have been shown to relate to outcome and function^17–19^.

Since automatic tractography methods are within reach to become feasible for routine data analysis, machine learning algorithms are of growing interest to handle the expansive amount of data^15^. However, feature selection is essential for the successful analysis of high-dimensional data^20^.

In order to safely resect high-grade gliomas, the expansion of our knowledge about functional white matter measurement is critical. This concerns surgical planning and navigation as well as the preparation of informed feature selection for machine learning in the future. We therefore analyzed the statistical relation of different white matter markers of the corticospinal tract (CST) with CST function and morphological CST affection.

## Methods

### Patients

This study was approved by the ethics committee of Leipzig University (297/21-ek) and was performed in accordance with the relevant guidelines and regulations. Informed consent was waived by the ethics committee due to the retrospective pseudonymized nature of the study. We retrospectively searched the database of our institution for all patients who were operated for a malignant glioma (WHO grade 3 and 4) and underwent preoperative MRI with DTI sequence between January 1, 2020 and December 31, 2021. Diagnostic criteria for tumor grading were based on integrated histomolecular classification and followed the WHO classification of 2016^21^. Inclusion criteria were an MRI including DTI within 7 days before surgery and age of at least 18 years.

Contralateral paresis was measured according to the MRC scale and recorded dichotomously as "yes" or "no" according to clinical neurological examination of the patient^22^.

### Imaging

MRI was conducted within seven days prior to surgery with a 3 T system (Ingenia, Philips, Eindhoven, Netherlands) using a single-shot echo-planar imaging diffusion tensor imaging (DTI) sequence (TR/TE = 7010/102 ms; FOV = 222 × 222 mm^2^; matrix 112 × 112; 50 slices without gap; slice thickness 2.7 mm; 32 non-collinear directions, b-value = 1000 s/mm^2^) and contrast-enhanced T1 weighted MPRAGE 3D dataset (TR/TE = 8.1/3.7 ms; FOV = 222 × 222 mm^2^; matrix = 512 × 512; 170 slices without gap, thickness 1 mm) using a dedicated head coil. The data was preprocessed with the dwipreproc^23^ command in MRtrix3 (www.mrtrix.org)^24^ including eddy correction and underwent a visual quality check. CST tractography was performed first manually with MRtrix3^17^, and also automatically in TractSeg. ^15^ Tractography parameters included an FA cutoff of 0.15, a maximum length of 250 mm, a minimum length of 60 mm, and 10^6^ seeds. The seed region was placed in the mesencephalon, and the target region in the internal capsule. An experienced neurosurgeon and an experienced neuroradiologist removed false-positive streamlines that were identified as collaterals to other tracts or showed clear signs of noise. CST affection was assessed in comparison to the contralateral CST by an experienced neuroradiologist and an experienced neurosurgeon, who were blinded for patient data, with: 0—unaffected (symmetric), 1—dislocated (asymmetric), 2—compressed (reduced volume), 3—infiltrated (contact or overlap with tumor). We recorded mean and median FA and streamline count from manual tractograms, and radiomic parameters from automatic tractograms^25^.

### Radiomics

Radiomics were calculated upon the volumes of the respective tractograms of the CST. The space was defined in the FA map, so that voxel based calculations used the respective FA. A complete description is available under https://pyradiomics.readthedocs.io/en/latest/features.html^25^.

#### FA energy

Energy is a measure of the magnitude of all voxel values within the respective volume, where a larger value marks a larger sum of the squares^25^.

$${\text{energy}}=\sum_{i=1}^{{N}_{p}}({\text{X}}(i)+c{)}^{2}$$

#### FA kurtosis

Kurtosis measures the shape of the distribution of values, where a lower kurtosis marks a high concentration of values in form of a peak near the mean.

$${\text{kurtosis}}=\frac{{\mu }_{4}}{{\sigma }^{4}}=\frac{\frac{1}{{N}_{p}}\sum_{i=1}^{{N}_{p}}({\text{X}}(i)-\overline{X}{)}^{4}}{{(\frac{1}{{N}_{p}}\sum_{i=1}^{{N}_{p}}({\text{X}}(i)-\overline{X}{)}^{2})}^{2}}$$

#### Maximum 2D diameter column

This is the maximum slice diameter of the CST in the axial plane. It is defined as the largest pairwise distance between volume surface mesh vertices in this plane.

#### Maximum 3D diameter

This is defined as the largest pairwise Euclidean distance between volume surface mesh vertices, also known as the Feret Diameter.

#### Minor axis length

This is the second largest axis of the enclosing ellipsoid and calculated with the largest principal component *λ*_*minor*_.

#### Elongation

Elongation reflects the ratio of the two largest principal components of the respective volume, where *λ*_*minor*_ and *λ*_*major*_ are the lengths of these components. It can take values between 0 and 1 and is defined as the inverse of true elongation for computational reasons.

$${\text{elongation}}=\sqrt{\frac{{\lambda }_{minor}}{{\lambda }_{major}}}$$

#### Surface area

Surface area is calculated with a mesh of triangles around the respective volume.

$$A=\sum_{i=1}^{{N}_{f}}{A}_{i}$$

$$\begin{array}{c}\begin{array}{c}{A}_{i}=\frac{1}{2}\left|{\text{a}}_{i}{\text{b}}_{i}\times {\text{a}}_{i}{\text{c}}_{i}\right|\end{array}\end{array}$$

#### Voxel volume

This volume is calculated through multiplication of the single voxel volume *V*_*k*_ with the number of voxels in the respective volume. It is less precise than mesh volume and is not used in further calculations.

$${V}_{voxel}=\sum_{k=1}^{{N}_{v}}{V}_{k}$$

#### Mesh volume

This volume is calculated from the mesh of triangles around the respective space by calculating the volume *V*_*i*_ of the tetrahedrons within that space defined by the origin *O* and the points *a*_*i*_, *b*_*i*_, and *c*_*i*_.

$$\begin{array}{c}\begin{array}{c}{V}_{i}=\frac{O{a}_{i}\cdot (O{b}_{i}\times O{c}_{i})}{6}\\ V=\sum\limits_{i=1}^{{N}_{f}}{V}_{i}\end{array}\end{array}$$

#### Surface volume ratio

This ratio is calculated with surface area *A* and mesh volume *V*.

$$\text{surface volume ratio}=\frac{A}{V}$$

### Statistics

Statistical analysis was carried out with SPSS Statistics 27 (IBM, Armonk, NY, USA). FA values of all tracts were tested for normal distribution after D'Agostino-Pearson yielding non-normal distribution in all cases. Correlations were tested by bivariate non-parametric Spearman's rank order for significance. P-values < 0.05 were considered statistically significant. Receiver-operating-characteristics analysis was applied to report area under the curve (AUC), sensitivity, and specificity for each variable. For further investigation, the analyzed variables were sorted by their respective p-value. Significant correlations were then plotted in a diagram with the dependent variable.

## Results

**Figure 1:**
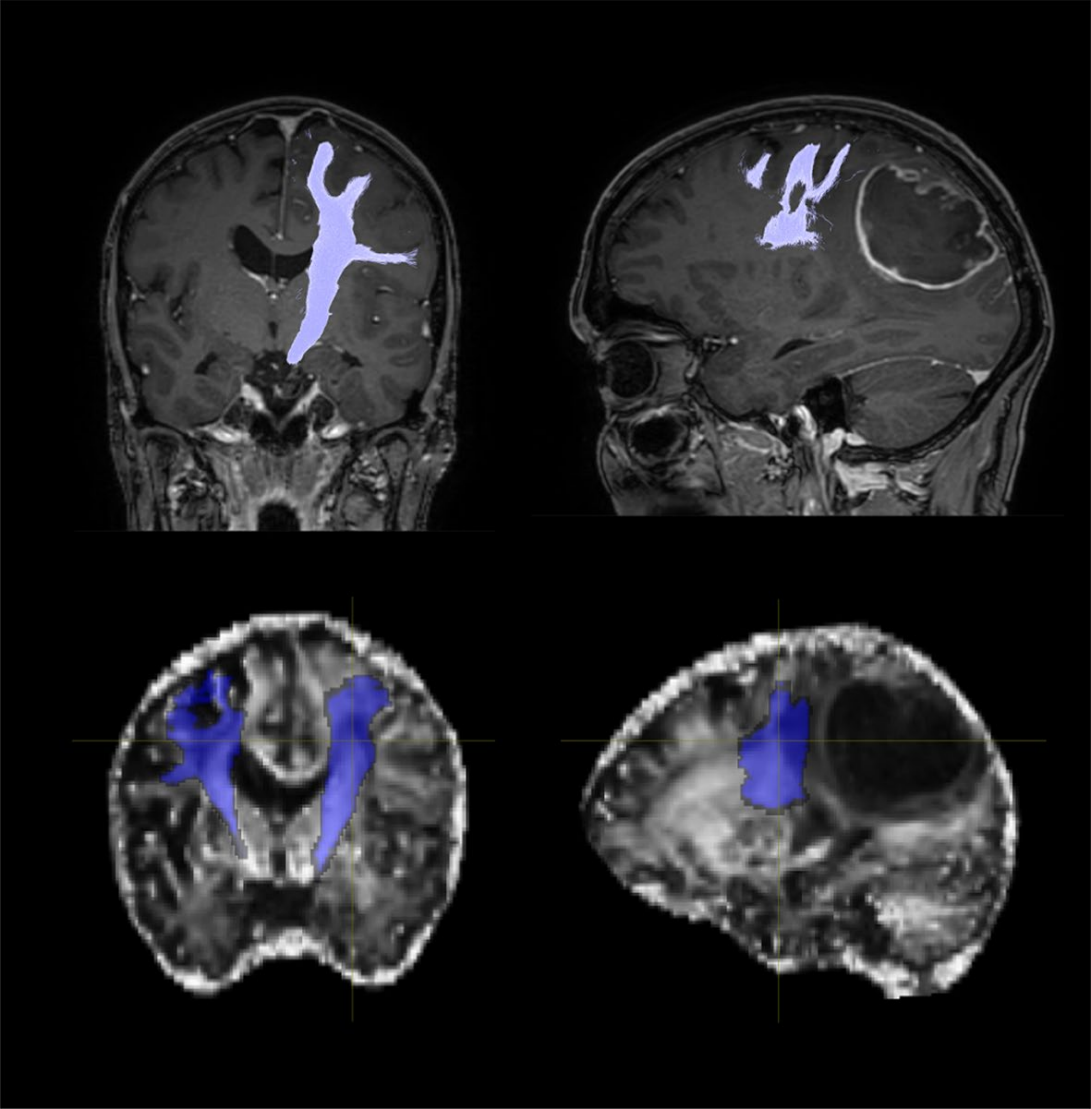
Example tractograms of the CST. Top row: Manual tractography result with MRTrix3 in coregistered contrast-enhanced T1 weighted MPRAGE 3D dataset. Bottom row: Automatic tractography result with TractSeg in a coregistered skull-stripped FA map. Left: coronal view. Right: sagittal view. Orientation in radiological convention.

Screening of 42 patients yielded 24 patients (42% female) with high-grade glioma (five grade 3, 19 grade 4). Median time between preoperative imaging and surgery was one day (range 1–5 days). Six patients suffered from preoperative contralateral paresis. Epidemiological data is shown in Table 1. Example tractograms are shown in Fig. 1. In morphological affection rating, CSTs were dislocated by glioma growth in four patients, compressed in six and considered to be infiltrated in five. In the remaining nine patients CSTs were marked unaffected.

**Table 1:** Baseline data.

Number of patients	24
Sex	
Male	14
Female	10
Mean age (years)	58 ± 3
Grade	
3	5
4	19
Paresis	
Yes	13
No	10
Mean CST FA	0.53 ± 0.03
Median CST FA	0.51 ± 0.04
CST FA energy	4199 ± 400
CST maximum 3D diameter (mm)	121 ± 2
CST surface area (cm^2^)	99.3 ± 5.5
CST voxel volume (ml)	24.5 ± 1.8
CST surface volume ratio (1/mm)	0.41 ± 0.02

**Figure 2:**
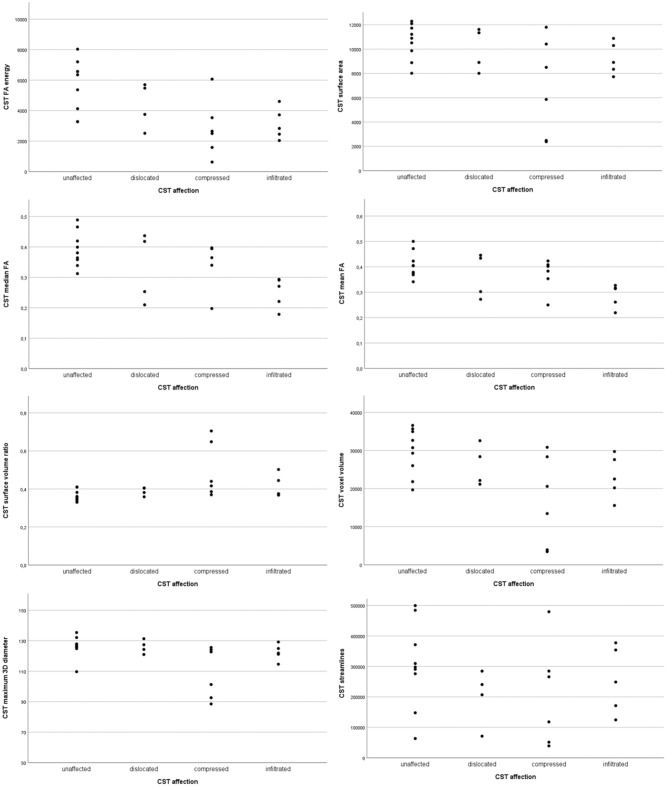
Scatter plots of significant correlations and streamline count with CST affection.

**Figure 3:**
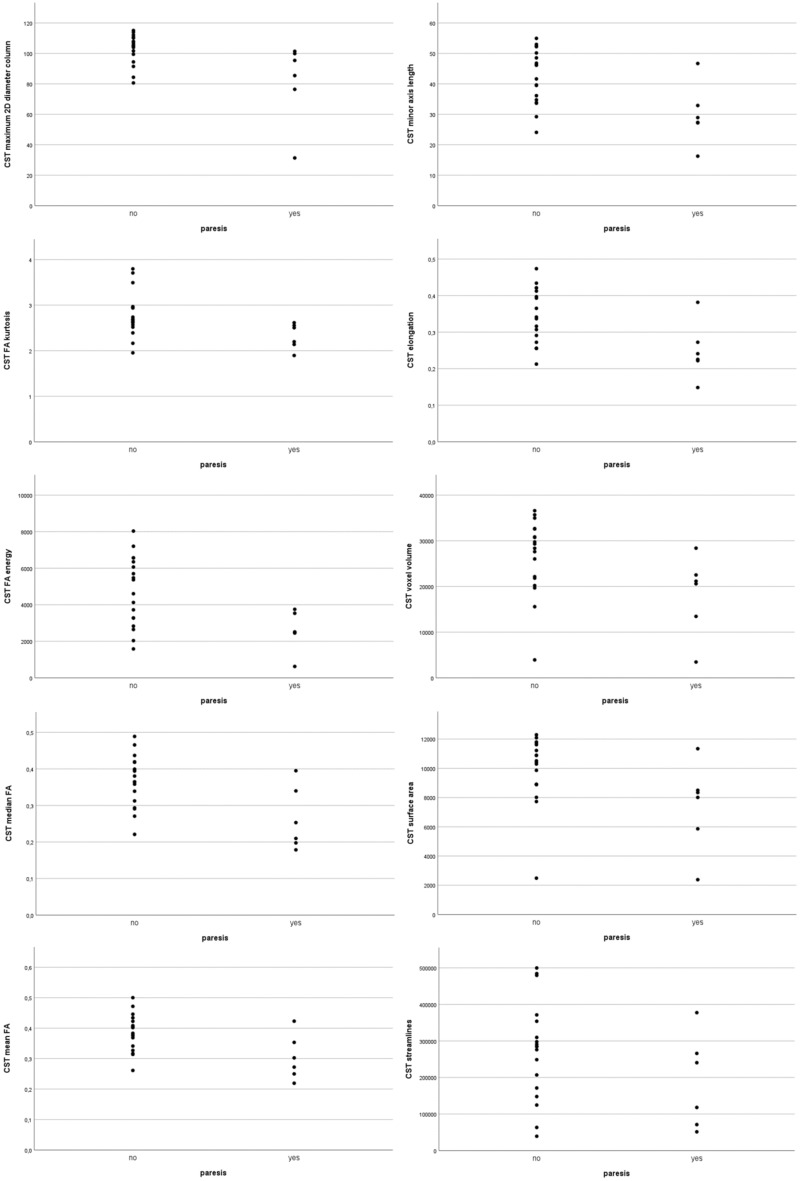
Scatter plots of significant correlations and streamline count with paresis.

CST affection correlated most significantly with energy (based on FA, p = 0.002), median and mean FA (p = 0.008), and surface-volume ratio (p = 0.014). Contralateral paresis showed a significant correlation with 2D column diameter (p = 0.005), minor axis length (p = 0.006), kurtosis (based on FA, p = 0.008), median FA (p = 0.015) and mean FA (p = 0.026). Correlations sorted by their respective p-values are shown in Tables 2 and 3. Scatterplots of significant correlations are shown in Figs. 2 and 3.

**Table 2:** Markers of radiological affection.

Variable	r	p	AUC	sensitivity	specificity
FA energy	– 0.605	0.002	0.853	0.769	0.818
Median FA	– 0.531	0.008	0.734	0.846	0.545
Mean FA	– 0.525	0.008	0.734	0.769	0.636
Surface volume ratio	– 0.495	0.014	0.804	0.545	1.0
Voxel volume	– 0.462	0.023	0.783	0.923	0.545
Maximum 3D diameter	– 0.459	0.024	0.811	0.692	0.909
Mesh volume	– 0.457	0.025	0.776	0.923	0.545
Surface area	– 0.414	0.044	0.755	0.538	0.909
Streamline count	– 0.226	0.289	0.608	0.615	0.636

Significant correlations (two-tailed) of radiomics and CST affection with the respective p-values. A negative correlation coefficient *r* indicates less affection.

**Table 3:** Markers of motor function.

Variable	r	p	AUC	Sensitivity	Specificity
Maximum 2D diameter column	0.556	0.005	0.852	0.722	1.0
Minor axis length	0.545	0.006	0.852	0.889	0.833
FA kurtosis	0.530	0.008	0.861	0.722	1.0
Elongation	0.514	0.010	0.824	0.944	0.667
FA energy	0.504	0.012	0.824	0.611	1.0
Median FA	0.492	0.015	0.833	0.944	0.667
Surface area	0.469	0.021	0.796	0.833	0.833
Mean FA	0.455	0.026	0.806	0.944	0.667
Mesh volume	0.447	0.029	0.787	0.566	1.0
Voxel volume	0.434	0.034	0.788	0.667	0.833
Streamline count	– 0.292	0.166	0.694	0.611	0.833

Significant correlations (two-tailed) of radiomics and CST function (paresis) with the respective p-values. A positive correlation coefficient *r* indicates better motor function.

## Discussion

This work analyzed the relation of different white matter parameters with clinical presentation in both radiological and clinical examination. The corticospinal tract (CST) was chosen due to its anatomical size and clinical importance in a large portion of neurological functions^26^. Damage to the CST is easily detectable and can be quantified in clinical presentation while being routinely demonstrated in MRI and more so in MRI tractography.

Furthermore, neurological deficits show significant correlations with overall survival in high-grade glioma, since a certain level of neurological function is always required for adjuvant treatment^27,28^. Neurological function and explicitly motor function is on the other hand important for maintaining quality of life in the treatment of high-grade glioma^29^.

The growing field of tractography studies motivates both clinicians and researchers to find better tools for patient evaluation and patient counseling by depicting and measuring different tracts or whole connectomes in patients with stroke, multiple sclerosis, or brain tumors^5,30,31^. The depiction of tract anatomy is becoming increasingly precise, which enables analyses of higher orders like connectomics^32^. However, surrogate variables of tract damage should be evaluated for their respective value before drawing conclusions from them in higher order analyses. Although FA has been shown to be a feasible marker of white matter integrity, streamline count is often used in the analysis of tractography or connectomes^33^.

In the presented analysis of high-grade glioma patients, we found no significant correlations of streamline count with radiological or clinical CST affection, which is well visible in the scatter plots of our data. Interestingly, different measurements of FA and CST volume showed different correlations with CST affection. Mean and median are easily applicable in both clinical and experimental setups, and have been demonstrated in our screening approach to indicate functional CST affection in imaging and neurological examination. This holds also true for mesh volume, voxel volume, surface area, and energy. While the latter is derived from FA values, it is interesting that its correlation with both radiological and clinical CST affection is stronger than mean and median. Since energy is the sum of the squared values of every voxel, it is volume-confounded, which may be the cause of its stronger correlation with CST integrity.

It is visible in the scatter-plots that voxel volume, maximum 3D diameter, mesh volume, and surface area correlate foremost with CST compression. This is to expect, since a reduced volume was the definition for compression in our data. Nevertheless, these variables correlate also with contralateral paresis, which points both towards intervariable correlations and overlapping effects of compression, infiltration, and function.

Surprisingly, three geometric variables correlate significantly only with paresis. Maximum 2D diameter column, minor axis length, and elongation seem to indicate CST function by its shape, independent from radiological CST affection. They may therefore be useful in automatic categorization of CST affection that is not easily assessable by expert interaction.

The fact that FA kurtosis is a strong indicator of CST function, but not radiological affection, is in line with previously published studies that mentioned postoperative FA in the CST to be more condensed around the mean. Interpreted together, it could indicate higher kurtosis and better motor function^16^.

## Limitations

The data was retrospectively collected. Further, different MRI scanners and different tractography algorithms may deliver different results, which remains to be disproven. Therefore, prospective multicenter studies with comparison of different scanners and algorithms would be needed for confirmation or rebuttal of comparability. Also, CST affection was measured manually, which leaves room for uncertainty of the measurement. However, we believe, that reproducibility is given by following the thorough description of affection rating. Nevertheless, further studies which may apply machine learning approaches should analyze larger cohorts.

## Conclusions

Our results confirm earlier studies that found FA to be a good indicator of white matter integrity and expand this finding for high-grade glioma. It is further shown that FA-derived radiomics like energy and kurtosis, as well as geometric radiomics like volume and elongation, are suitable indicators of tract integrity and can be used in further research on the functional relevance of white matter tracts. Streamline count should not be used for functional analysis.

## Data Availability

Data sets generated during the current study are available from the corresponding author on reasonable request.
